# Phytochemicals: a promising approach to control infectious bursal disease

**DOI:** 10.3389/fvets.2024.1421668

**Published:** 2024-06-11

**Authors:** Ifrah Tahir, Abdullah F. Alsayeqh

**Affiliations:** ^1^Department of Parasitology, University of Agriculture, Faisalabad, Pakistan; ^2^Department of Veterinary Preventive Medicine, College of Veterinary Medicine, Qassim University, Buraidah, Saudi Arabia

**Keywords:** plants, poultry, antioxidant, immune response, virus, polyphenol

## Abstract

Infectious bursal disease (IBD) is one of the dangerous diseases of poultry that affects the bursa of Fabricius, which is an important organ of the bird’s immune system. IBD virus is resistant to many drugs, making its control difficult. Vaccination of IBD is in practice for a long time worldwide to control IBD, but secondary issues like vaccine failure and lower efficacy lead to their reduced use in the field. Multiple medicines are currently used, but the phytochemicals have emerged as promising agents for controlling IBD. The drugs to be developed should possess direct antiviral properties by targeting viral entry mechanisms, enhancing the host immune response, and inhibiting viral protein synthesis. Phytochemicals have potential to contribute to food security by minimizing the possibility of disease outbreaks and ensuring that consumers worldwide obtain healthy poultry products. It has been now claimed that direct and indirect activities of phytochemicals can be effective in the control of IBDV. Although available evidence suggest that the phytochemicals can contribute in controlling occurrence IBDV, there is a definite need of focused studies to gain more insight and develop rational strategies for their practical use. This review highlights the disease caused by IBDV, inhibition of viral replication, boosting the immune system, disruption of viral membrane, and important phytochemicals showing antiviral activities against IBDV.

## Introduction

Poultry is a significant source of income and food that is essential for food security and global agriculture (1, 2). Poultry products like chicken meat and eggs are accessible sources of protein. Additionally, they are a good source of vital nutrients for human health, such as vitamins and minerals (3, 4). The largest and most significant source of animal protein for human consumption is the poultry industry (5, 6). More than 130 million metric tons of poultry meat and more than 86.67 million metric tons of eggs were produced worldwide in 2020 (7). Bacteria (*Mycobacterium tuberculosis*, coliform bacillary), ectoparasites and endoparasites, fungal pathogens, and eleven virus species can spread diseases horizontally and vertically in poultry (8, 9). One of the main reasons for financial losses in the global chicken industry is viral outbreaks (10, 11). Zootechnical performances, including body weight gain, feed conversion ratio, feed intake, and the quality of the eggs and meat produced, are significantly impacted by viral outbreaks in the poultry industry (12, 13). Avian influenza virus (AIV) (14), Newcastle disease virus (NDV) (15), Marek’s disease virus (MDV), infectious laryngotracheitis virus (ILTV), infectious bursal disease virus (IBDV) and infectious bronchitis virus (IBV) are the major pathogens that cause diseases in poultry (16).

IBDV is a highly contagious, positive sense, single-stranded, and RNA-based gammacoronavirus from the family of coronaviridae (17). IBD is a widespread infectious disease caused by a virus that infects young chickens and weakens their immune system (18, 19). It is caused by a birnavirus and mostly affects the bursa of Fabricius, an important organ in poultry for the growth of their immune systems (20, 21). Multiple IBDV serotypes are frequently observed co-circulating on farms, with little cross-protection from the vaccination strain against unrelated field strains. Multiple other species of IBDV cause diseases in various species of birds (Table 1).

**Table 1:** Infectious bursal disease, their host range, and disease in the birds.

IBDV	Host range	Disease	Disease signs	References
Variant IBDV	Turkeys, chickens	Variant IBD	Lethargy and depression	(22)
—	Pheasants, quails	—	Diarrhea	(23)
—	Pigeons, ducks	—	Reduced feed intake and weight loss	(24)
—	Partridges, guinea fowl	—	Immunosuppression leads to secondary infections	(25)
IBDV serotype 1	Chickens	IBD	Dehydration, reduced feed intake and weight loss, watery or bloody diarrhea, depression and lethargy	(26)
IBDV serotype 2	Turkeys	Asymptomatic or mild disease	No apparent clinical signs, subclinical infection	(27)
Very virulent IBDV (vvIBDV)	Chickens (specific strains)	(Viral hemorrhagic disease)	Immunosuppression leads to secondary infections, diarrhea, often with blood, sudden death, depression and huddling behavior	(28)

IBDV has spread over South East Asia, the Middle East, Europe, Africa, and Latin America, causing high mortality in chickens (29, 30). IBDV is extremely infectious and spreads at a high rate in poultry (31). Young chicks are especially susceptible to IBDV, which causes immunosuppression and mortality often between 3 and 6 weeks of age (32, 33). The virus may remain in the environment for several weeks and is excreted in the feces of infected birds (34, 35). Although indirect transmission via contaminated feed, water, equipment, or employees and direct contact between sick and vulnerable birds is the main source of infection” the primary mode of infection is indirect transmission through contaminated water, feed, equipment, or personnel, as well as direct contact between infected and susceptible birds (15, 36). The virus may spread between farms through rodents, wild birds, and insects as carriers (37). The virus enters in a chicken body through the digestive and respiratory system where it infects the dendritic cells and macrophages that are present in the mucosal lining of the digestive and respiratory tract (38). After penetration into the cell, IBDV replicates quickly, facilitating its spread through the body via blood circulation and the lymphatic system. Afterward, the virus infects B-lymphocytes, vital for immune system development (39). IBDV destroys the B-lymphocytes, decreasing antibody production, and leading to immunosuppression (40). The circulating B-cells in the secondary lymphoid tissues and lymphoid follicles in the bursa of Fabricius are both destroyed by the virus. As a result, fewer antibodies are produced, cell-mediated immunity is compromised, and the body is more vulnerable to other diseases (41). The injured birds are more susceptible to secondary infections and immunosuppressive effects can last for several weeks (42). Chicken with immunosuppression and low immunity are more susceptible to disease and vaccine failure. Both layer and broiler flocks are susceptible to IBD which can result in high mortality rates, less production, and substantial financial loss for producers (43). The pathogenicity of IBDV can vary depending on the immune status, age of the infected birds, and the viral strain (44). The symptoms of IBD include lethargy, depression, exhaustion, decreased appetite, watery diarrhea, immune depression, less energy, and a drop in feed consumption (45). With decreased productivity, higher mortality rates, veterinary costs, decreased egg production, and trade restrictions, IBDV can significantly affect the economics of poultry production. This critical problem emphasizes the necessity for researchers to develop strategies to reduce IBDV. One of the most popular solutions for treating IBDV on a commercial level is vaccinations. There are several commercially available IBDV vaccinations (46). Vaccines are being developed to target various strains of the IBDV and are effective against multiple species of the parasite. Modified-live vaccine (MLV), IBD vectored vaccine (vHVT13), live attenuated vaccines, inactivated vaccines, immune-complex (Icx), and live recombinant vaccines expressing the capsid (VP2) antigen of IBDV vaccines are being used in routine farming (47). IBD vaccines may provide immunity, but some problems limit their use. Some commonly available vaccinations are no longer effective in protecting IBDV in many commercial poultry farms (48). The apparent inability to prevent IBDV infections through vaccination may occasionally be caused by improper vaccine virus administration, antigenic variations among the viruses, insufficient potency of the live attenuated vaccine virus, or interaction between the vaccine virus and any residual maternally derived antibodies (49). The traditional strain vaccine was not protective against variant IBDV strains. Additionally, IBDV vaccines provide only transient protection and require repeated application, especially in layer and breeder chicken (50). The expensive cost of the IBDV vaccine restricts its use in poultry. The virus is challenging to control since it can endure for a long time in the environment. Finding a suitable substitute to combat IBD is necessary due to these problems. Several approaches are being investigated, for the prevention and management of IBD, including the use of amino acids, organic acids, and their derivatives (51). Phytochemicals are also proposed alternatives for controlling IBD, as they are gaining attention from researchers for their antifungal (3), anti-infectious, anti-inflammatory, antioxidant, and immunomodulatory properties (52, 53). These investigations have generated valuable insights into how plants can efficiently mitigate various types of IBD (54). However, there is a need to further investigate the pharmacological properties and the mechanism of action of these botanicals. In this review, we have summarized the effective agents of phytochemicals and their modes of action, as well as the properties that make them beneficial for use against IBD.

### Pathology and methods for controlling IBD

**Figure 1:**
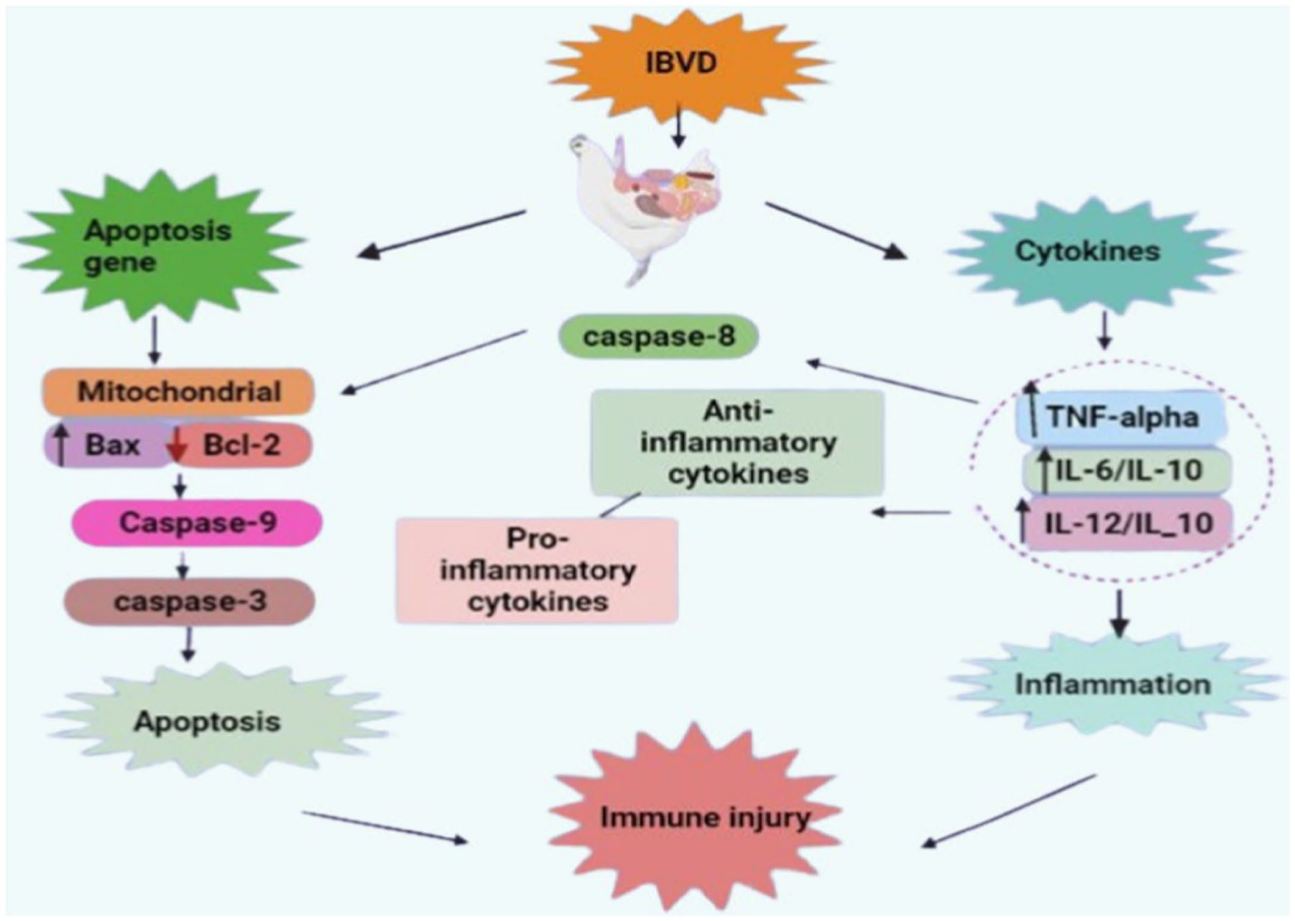
Pathogenesis of infectious bursal disease (retrieved from https://app.biorender.com/).

It is important to identify the points at which IBD can be effectively controlled before reviewing the plants and plant products used to control IBD. IBDV is transferred by infected macrophages to the bursa of Fabricius and experiences intracytoplasmic replication in IgM+ B lymphocytes (55). The stimulation of macrophages increases interferon- (IFN-) production in the bursa of Fabricius (56). Nitric oxide and other pro-inflammatory cytokines, including interleukin-6 (IL-6), are released simultaneously (57). The bursal lesions developed as a result of these cytokines. Additionally, the healthy B-cells around IBDV-infected cells may undergo apoptosis as a result of the IFN-γ produced during the infection (58) (Figure 1).

The bone marrow and caecal tonsils can help in the later replication of IBDV. According to flow cytometric studies, the IgM+ of the B-cell population is significantly lower than the IgA and IgG. IBDV has been reported to be generally unaffected by the mesenchymally derived reticular cells seen in the germinal center, bursal cortex, red pulp of the spleen, and periarteriolar lymphoid sheaths (59). The absence of B-cell proliferative environment is assumed to be the cause of the elimination of bursal follicular dendritic cells in IBDV infection. Some points where IBDV can be controlled by recognizing the stages of this cycle and its related diseases. To prevent IBD, strict hygiene standards and vaccination with traditional live, attenuated, and inactivated viral vaccines have been utilized (60). It is challenging to remove the persistent IBDV particles from the farm because the virus endures for 52 days in feed and water and 122 days in poultry farms (47). It is expensive to control IBDV in infected chickens and prevent the spread of the disease to other flocks. As a result, vaccination is still the preferred strategy for controlling IBD. Even with strict immunization procedures, vaccine failures still happen as a result of the virus evolution (61). To reduce the immunosuppressive effects of IBDV, it is crucial to prevent infection at a young age, but parent stock can be immunized to achieve this (25). Many researchers have explored the use of phytochemicals for controlling IBD. The effectiveness of the plant in combating the disease depends on its active constituents.

### Phytochemicals

Phytochemicals, usually referred to as phytonutrients, are physiologically active substances that are present in plants in their natural state (62, 63). Fruits, vegetables, cereals, and other plant-based foods receive their colors, flavors, and aromas from phytochemicals (64). There are 1,000 different phytochemicals, each with its own special qualities and purposes (65).

**Figure 2:**
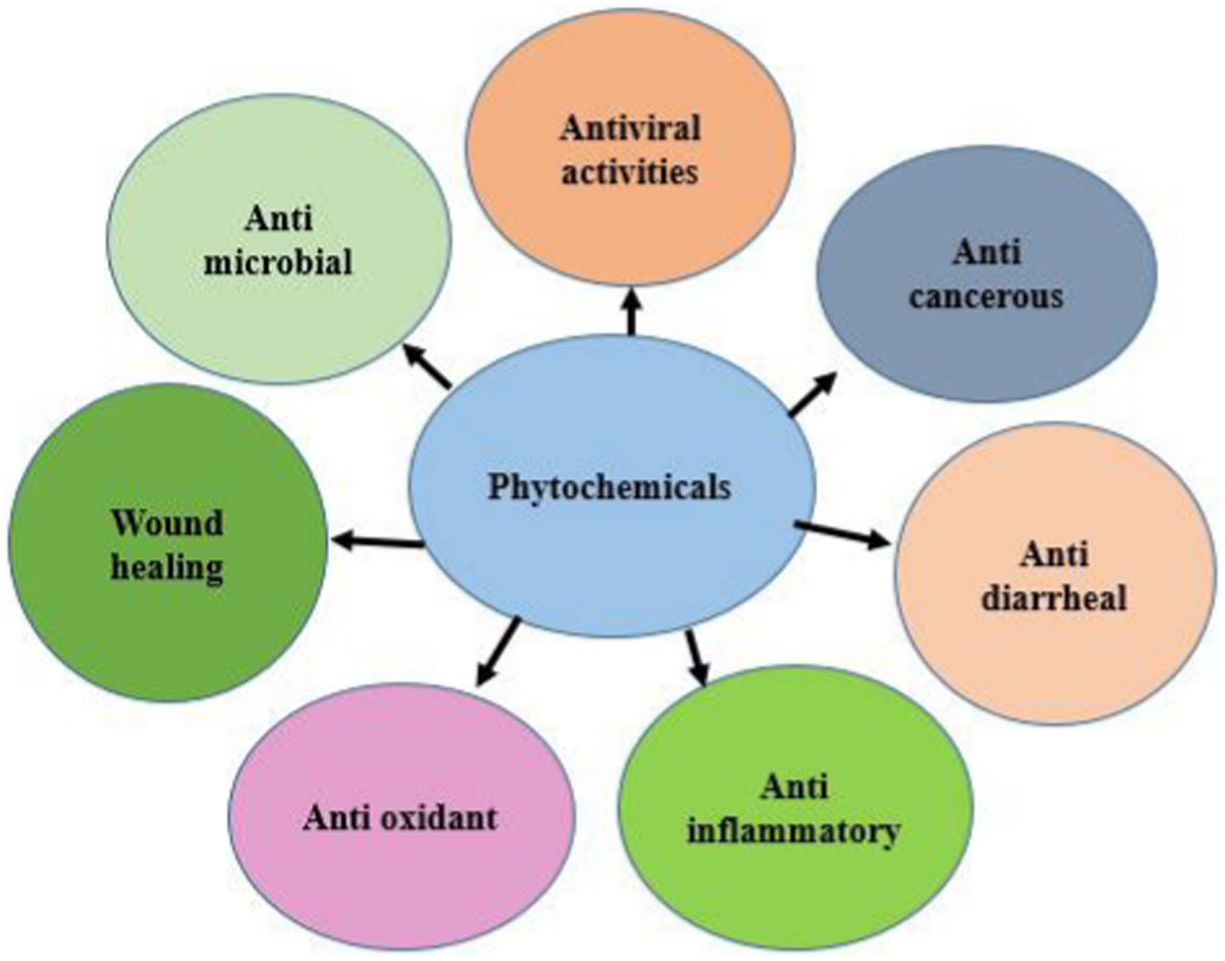
Therapeutics properties of phytochemicals (retrieved from https://app.biorender.com/).

Phytochemicals have a variety of bioactivities, including antiviral, antibacterial, antiparasitic, antioxidant, antivenin, larvicidal, antifungal, anti-inflammatory, anti-diabetic, anti-amoebic, and wound healing properties (Figure 2) (66). Numerous phytochemicals have antioxidant characteristics that can aid in preventing cellular damage and oxidative stress which are factors in a variety of health issues (67). In the body, dangerous free radicals that can harm cells and cause chronic inflammation and disease are neutralized by antioxidants (68). Additionally, phytochemicals control gene expression and have an impact on the activity of numerous proteins and enzymes in the body (69). Various phytochemicals can target different physiological pathways and activities that can have a wide range of impacts on health and the prevention of disease (70). Currently, the value of herbal medicine is promising. It is often employed in the treatment of numerous illnesses, even those that are incurable (71). The use of phytochemicals as natural alternatives to medicinal products for a variety of health issues has gained popularity in recent years (54). Different plants have been examined for their activity against IBD, which has been mentioned in Table 2.

**Table 2:** Exploring the efficacy of bioactive phytochemicals in controlling infectious bursal disease.

Plant	Plant part	Phytochemicals	Properties	Mode of action	References
*Curcuma longa*	Rhizome	Curcumin	Immunomodulatory, antiviral	Reduces viral load, inhibits IBDV replication	(72, 73)
*Allium sativum*	Bulbs	Alliin, allicin	Antiviral, immunomodulatory	Enhances immune response, inhibits IBDV replication	(74, 75)
*Allium cepa*	Bulbs	Quercetin	Antioxidant, antiviral	Inhibits replication, reduces IBDV-induced oxidative stress	(76)
*Vitis vinifera*	Fruit	Resveratrol	Immunomodulatory, antiviral	Enhances immune response, suppresses IBDV replication	(77)
*Camellia sinensis*	Leaves	Epigallocatechin	Antiviral, immunomodulatory	Reduces viral load, inhibits IBDV attachment and entry	(78)
*Thymus vulgaris*	Leaves	Luteolin	Antiviral, anti-inflammatory	Inhibits IBDV replication, reduces inflammation	(79)
*Goldenseal*	Rhizomes	Berberine	Immunomodulatory, antiviral	Interferes with IBDV replication, enhances immune response	(80)
*Zingiber officinale*	Rhizome	Gingerol	Antioxidant, antiviral	Protects against oxidative damage, inhibits IBDV replication	(81)
*Punica granatum*	Fruit	Ellagic acid	Immunomodulatory, antiviral	Reduces IBDV replication, modulates the immune response	(82)
*Camellia sinensis*	Leaves	Catechins	Antioxidant, antiviral	Reduces oxidative stress, impairs IBDV replication,	(83, 84)
*Coffea arabica*	Seeds	Caffeic Acid	Antioxidant, antiviral	Inhibit viral replication, reduce oxidative stress	(85)
*Quercus* spp.	Bark	Ellagitannins	Antiviral, antioxidant	Reduce oxidative stress, inhibit viral attachment	(86)
*Panax ginseng*	Leaves	Ginsenosides	Immunomodulatory, antiviral	Boost immune response, disrupt viral membrane	(87)
*Citrus limon*	Peel of lemons	Limonene	Immunomodulatory, antiviral	Enhance immune response, inhibit viral replication	(88)
*Thymus vulgaris*	Leaves	Thymol	Antimicrobial, antiviral	Reduce microbial load, inhibit viral replication	(89)
*Hordeum vulgare*	Grain	β-glucans	Antioxidant, immunomodulatory	Reduce oxidative stress, enhance immune response	(90)

### Mechanisms of action of phytochemicals

**Figure 3:**
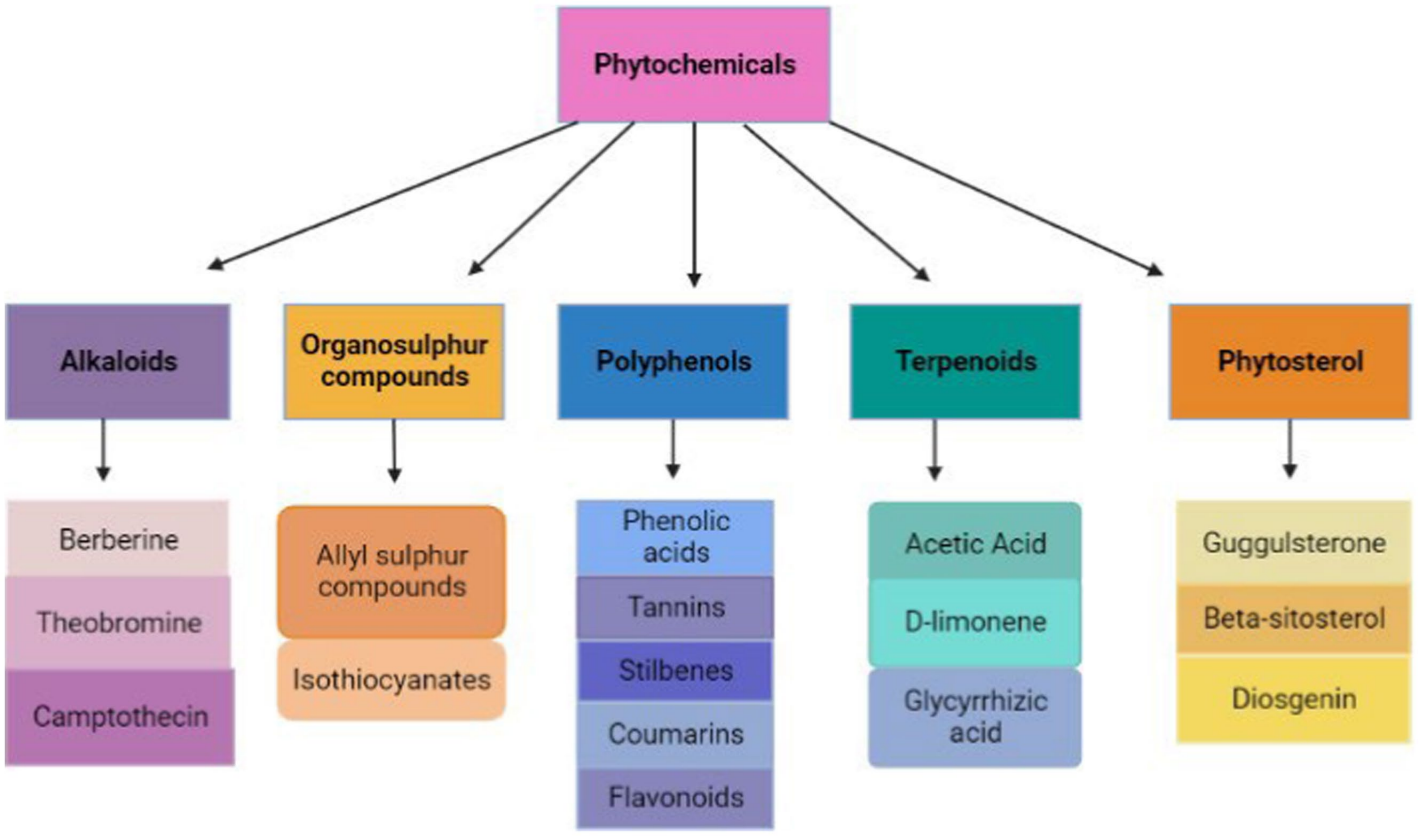
Types of phytochemicals (retrieved from https://app.biorender.com/).

Phytochemicals can be used in the treatment of IBD due to their anti-inflammatory, antiviral, and immunomodulatory effects (91). Certain phytochemicals, including alkaloids, flavonoids, and polyphenols, prevent IBDV from replicating by preventing the virus from entering host cells (Figure 3) (86). Additionally, phytochemicals may alter the reaction of the host immune system to viral infection (92). Some flavonoids can increase the production of cytokines, which are essential for the immune response, while other flavonoids may suppress the production of pro-inflammatory cytokines, which can result in tissue damage (93). Essential oils and other phytochemicals have been demonstrated to strengthen the host immune system by boosting the generation of natural killer cells and other immune cells, which affect the strengthening of the host’s defense against viral infection (94). Phytochemicals can also use their antiviral benefits by lowering oxidative stress, which is a characteristic of viral infections (95). Phytochemicals with antioxidant characteristics, such as flavonoids and polyphenols can shield host cells from harm by reactive oxygen species produced during viral infection (96).

### Botanical compounds for controlling infectious bursal disease

#### Flavonoids

Flavonoids are a subclass of phenolic compounds, distinguished by having two phenolic rings joined by a three-carbon bridge (97). Flavonoids have two aromatic rings (A and B rings) joined by a heterocyclic ring (C ring) as its fundamental structure (98). The subclassification of flavonoids includes flavanols, flavanones, flavones, isoflavones, catechins, and anthocyanins (99). One of the main compounds that have been investigated for its antibacterial properties is flavonoids (100). Flavonoids and their potential chemo-preventive bioactivities have received a lot of interest in the past two decades, particularly their antiviral characteristics in viral infectious diseases (101). Currently, flavonoids have been investigated against a variety of RNA and DNA viruses. In humans, they exhibit a variety of physiological effects, including antiviral, cytotoxic, anti-inflammatory, antiallergic, antibacterial, antioxidant, and anticancer activities. Flavonoids are pleiotropic substances, which means that their functional groups can interact with a variety of cellular targets and obstruct a variety of pathways (102). They also lack systemic toxicity, and their capacity to work in concert with conventional medications has largely been established (103). These characteristics make flavonoid candidates to disrupt the viral life cycle. Flavonoids are chemicals that boost the immune system by increasing IgM and IgG antibody production, which supports the humoral immune system of broilers (104). Flavonoids stimulate the synthesis of cytokines, particularly interferon, by activating macrophages. By decreasing bursal lesions and viral protein expression, they are particularly efficient against infectious IBDV in broilers (105). Many researchers have suggested that flavonoids could be used to control IBDV because of these properties (104).

#### Saponins

Saponins are chemical compounds found in a wide range of plants, including many therapeutic herbs (106). A variety of biological properties, including anti-inflammatory, antibacterial, antiviral, anticancer, antifungal, and immune modulatory actions, are present in saponins (107). Saponins are presently receiving attention for their potential therapeutic applications due to their diverse actions (108). Saponins interact with cholesterol in the intestines to produce complexes that prevent absorption (109). Saponins have antioxidant qualities that can aid in oxidative stress defense and improve overall health. Saponins can serve as immunoadjuvants, compounds that strengthen the reaction of the immune system to antigens (110). Saponins may help to regulate or lessen the effects of IBD by enhancing the immune system (111). Saponin increases the numbers of IgA+ cells and Intraepithelial Lymphocytes in the ileum, duodenum, and jejunum, while bursal lesions decreased in poultry (87). Plants that contain saponins also serve as astringents, lowering surface tension within the body and facilitating nutrition uptake by cells (112). These actions have led to the use of numerous saponin-containing plants in the treatment of IBD (113).

#### Terpenes and terpene derivatives

Terpenes are secondary metabolites found in plants that have an isoprene (2-methylbuta-1,3-diene) unit as their carbon backbone (114). There are about 40,000 distinct molecules of primary and secondary metabolism that belong to the largest class of phytochemicals known as terpenoids (115). Terpenes undergo various metabolic changes, such as oxidation and rearrangement, to produce terpenoids (116). These biochemical changes form terpenoids of oxygenated compounds of terpenes such as ketones, alcohols, acids, esters, ethers, and aldehydes. The only adequate volatile components of essential oils are sesquiterpenoids, hemi terpenoids, and mono terpenoids (117). These substances have the ability to integrate into phospholipid membranes because they are lipophilic (118). Because of their structure, terpenoids are more effective than synthetic medications at incorporating lipid layers into lipid layers or disrupting the important sterol metabolism of numerous pathogens (119). Terpenes can prevent viral DNA replication and interfere with virus adhesion to cellular membranes (120). These substances prevented viral replication by blocking the synthesis of viral structural proteins and the genes that code for the viral nucleocapsid protein, viral membrane protein, and viral spike (121). Research describes that terpenes and essential oils rich in terpenes are effective against IBD (86, 122).

#### Alkaloids

A significant class of naturally occurring organic compounds known as alkaloids makes up the majority of the largest group of phytochemicals (123). Alkaloids are distinguished by having nitrogen atoms, which gives them their alkaline characteristics and therapeutic effects (124). The majority of alkaloids are produced through the synthesis of amino acids like tryptophan, tyrosine, phenylalanine, ornithine, and lysine (125). The alkaloids have been shown to have anti-fungal, anti-inflammatory, and antibacterial effects (126, 127). Several alkaloid phytochemicals have the ability to inhibit and interact with viruses. To restrict access to host cells, they can obstruct attachment or viral fusion with the receptors on the surface of the host cell (128).

They can also interact with protein, viral RNA and DNA synthesis, and viral protein assembly (129). By concentrating on transport pathways, they can also stop viruses from invading other normal cells in the host (130). These characteristics have made alkaloids the preferred compound to be searched for their medical use against IBD. Multiple alkaloids have been used by researchers and found effective in controlling IBD (131).

#### Sulfur compounds

Sulfur has anti-inflammatory, antibacterial, antifungal, and antioxidant effects (132). They can boost the overall health of the immune system by assisting in the neutralization of free radicals and the reduction of oxidative stress (133). By affecting the generation and function of immune cells including macrophages and lymphocytes, sulfur components have been identified to alter immunological responses (134). They may increase immune cell activity and proliferative capacity, strengthening the immunological response (135). Sulfur components have been found to regulate immune responses by affecting the activity and production of immune cells such as macrophages and lymphocytes (136). They may increase immune cell activity and proliferation, resulting in a stronger immunological response (137). Allicin, S-allyl cysteine, Diallyl trisulfide, and Diallyl disulfide are examples of sulfur compounds. Allicin has an immune-stimulatory effect on poultry (138). Allicin, alliin, and other sulfur-containing substances have a potently stimulating effect on poultry immune systems (139). Multiple sulfur compounds have been used by researchers and found effective in controlling IBD (140, 141).

#### Vitamins

Vitamins are produced from the fruit and leaves of plants and play an important role in boosting the immune system leading to the control of major pathogenic diseases. Vitamin C boosts cellular and humoral responses, as well as a birds resistance to infections including IBD illnesses (142). Vitamin C improved the antibody production against IBDV in poultry. These results can be attributed to the increased activity of B lymphocytes and T lymphocytes in poultry (143). Various parameters of the immune system including specific antibody production, resistance to infections, numbers of antibody-producing cells, phagocytic index, and *in vitro* mitogenic reactions to lymphocytes, are modified by vitamins and not with antioxidant compounds (144). ARG, an essential amino acid for poultry, and an important antioxidant, influence positively both the cell-mediated and humoral immune responses of poultry (145). Arginine strengthens the immune system against bacterial and viral infections (146). Vitamin E enhances the immune response by decreasing prostaglandin E2 production, boosting macrophage phagocytic function, and T-cell proliferation, enhancing IL-2 production, and increasing interleukin-1 secretion by macrophages (147). Both Arginine and vitamin E can enhance immunological responses and may have an impact on resistance to disease (148, 149).

## Conclusion

Phytochemicals derived from plants are the largest source of production of medicines. Many years ago, medicinal plants have been used for the control and prevention of disease. In this review, we observed that phytochemicals have the potential to control IBD. Various compounds extracted from plants showed excellent results against IBD. Phytochemicals show potential treatment for IBD in poultry.

Further focused research aimed at identifying promising derivatives having significant targeted effects on IBDV is highly required to develop practical and rational strategies for use of phytochemicals.

## Author contributions

IT: Conceptualization, Data curation, Formal analysis, Funding acquisition, Investigation, Methodology, Project administration, Resources, Software, Supervision, Validation, Visualization, Writing – original draft, Writing – review & editing. AA: Conceptualization, Data curation, Formal analysis, Funding acquisition, Investigation, Methodology, Project administration, Resources, Software, Supervision, Validation, Visualization, Writing – original draft, Writing – review & editing.
